# *Agromyces silvae* sp. nov., *Rathayibacter soli* sp. nov., and *Nocardioides terrisoli*sp. nov., Isolated from Soil

**DOI:** 10.4014/jmb.2404.04007

**Published:** 2024-05-23

**Authors:** Hyosun Lee, Dhiraj Kumar Chaudhary, Dong-Uk Kim

**Affiliations:** 1 Department of Biological Science, College of Science and Engineering, Sangji University, Wonju 26339, Republic of Korea; 2 Department of Microbiology, Pukyong National University, Busan 48513, Republic of Korea

**Keywords:** *Agromyces silvae* sp. nov., *Rathayibacter soli* sp. nov., *Nocardioides terrisoli*sp. nov., soil, *Microbacteriaceae*, *Nocardioidaceae*

## Abstract

Three Gram-stain-positive, aerobic, rod-shaped, and non-motile bacteria, labelled as W11^T^, SW19^T^, and YR1^T^, were isolated from soil, and performed their polyphasic taxonomic investigation. The phylogenetic and 16S rRNA gene sequence analysis showed that strains W11^T^, SW19^T^, and YR1^T^ belonged to the genera *Agromyces*, *Rathayibacter*, and *Nocardioides*, respectively. Strain W11^T^ was closely affiliated with *Agromyces cavernae* SYSU K20354^T^ (98.1%), strain SW19^T^ showed the closest affiliation with *Rathayibacter rubneri* ZW T2_19^T^ (97.0%), and strain YR1^T^ was most closely related to *Nocardioides marmorisolisilvae* KIS18-7^T^ (98.0%). The genome sizes of strains W11^T^, SW19^T^, and YR1^T^ were 4,181,720 bp, 4,740,677 bp, and 4,228,226 bp, respectively, with DNA G+C contents of 70.5%, 64.2%, and 69.7%, respectively. Average nucleotide identity and digital DNA–DNA hybridization values of W11^T^, SW19^T^, and YR1^T^ with their respective reference species were <79.6% and <23.6%, respectively. The predominant cellular fatty acids detected in strain W11^T^ were anteiso-C_15:0_, iso-C_16:0_, and anteiso-C_17:0_. In strain SW19^T^, they were summed feature 9 (C_16:0_ 10-methyl and/or iso-C_17:1_ω 9c), anteiso-C_17:0_, and anteiso-C_15:0_. Strain YR1^T^ exhibited C_18:1_ω 9c, C_18:0_ 10-methyl, TBSA, and anteiso-C_15:0_ as its major cellular fatty acids. Overall, the polyphasic taxonomic comparisons indicated that strains W11^T^, SW19^T^, and YR1^T^ represent novel species within the genera *Agromyces*, *Rathayibacter*, and *Nocardioides*, respectively. Accordingly, we propose the names *Agromyces silvae* sp. nov., with the type strain W11^T^ (=KCTC 49818^T^ =NBRC 115999^T^), *Rathayibacter soli* sp. nov., with the type strain SW19^T^ (=KCTC 49860^T^ =NBRC 116108^T^), and *Nocardioides terrisoli*sp. nov., with the type strain YR1^T^ (=KCTC 49863^T^ =NBRC 116165^T^).

## Introduction

The genus *Agromyces*, classified within the family *Microbacteriaceae* of the phylum *Actinomycetota*, was originally introduced by Gledhill and Casida with *Agromyces ramosus* as its type species [1]. Currently, *Agromyces* comprises 43 species with the correct names (accession date: March 25, 2024; https://lpsn.dsmz.de/genus/agromyces). Similarly, the genus *Rathayibacter*, which also belongs to the family *Microbacteriaceae* in phylum *Actinomycetota*, was delineated by Zgurskaya et al. with *Rathayibacter rathayi* as the type species [2]. Currently, *Rathayibacter* includes a total of 9 species (accession date: March 25, 2024; https://lpsn.dsmz.de/genus/rathayibacter). Furthermore, the genus *Nocardioides*, belonging to the family *Nocardioidaceae* also within the phylum *Actinomycetota*, was proposed by Prauser, with *Nocardioides albus* as its type species [3]. As of the time of writing of this study, *Nocardioides* accommodates 166 valid species (accession date: March 25, 2024; https://lpsn.dsmz.de/genus/nocardioides). The members of genera belonging to *Actinomycetota* have been identified from diverse habitats, such as soil [4], cave [5], gut [6], sediment [7], fermented foods [8], wetland [9], and groundwater [10]. Several strains belonging to these genera are also derived from plant materials and are widely known as endophytic actinobacteria [11-14]. In this study, we performed taxonomic investigations of three strains (W11^T^, SW19^T^, and YR1^T^) isolated from soil. Our findings indicate that strains W11^T^, SW19^T^, and YR1^T^ represent novel species within the genera *Agromyces*, *Rathayibacter*, and *Nocardioides*, respectively.

## Materials and Methods

### Isolation of Strains

Strains W11^T^, SW19^T^, and YR1^T^ were isolated from soil samples collected from different sites of the Republic of Korea (W11^T^: 36°47'30.1"N 127°29'47.0"E, SW19^T^: 37°05'57.1"N 127°02'19.0"E, and YR1^T^: 36°30'20.2"N 127°01'13.1"E). The strains were obtained using the standard dilution plating method with R2A media (MB Cell, Republic of Korea). After the diluted samples were plated, the petri dishes were aerobically incubated at 25°C for 7 days. After incubation, individual colonies were chosen and streaked multiple times on R2A agar. Three distinctly pure colonies, designated W11^T^, SW19^T^, and YR1^T^, were obtained and temporarily maintained at 4°C until taxonomic analysis was completed. Subsequently, all strains were preserved in glycerol stock at -80°C.

### 16S rRNA and Phylogenetic Analysis

Genomic DNA from the W11^T^, SW19^T^, and YR1^T^ strains was extracted using the HiGene Genomic DNA Prep Kit (BIOFACT, Republic of Korea). The 16S rRNA gene was amplified, sequenced, and analyzed, as described previously [15]. The primers used for 16S rRNA gene amplification were 27F and 1492R [16]. The 16S rRNA gene sequences were analyzed on the EzBioCloud database server [17]. Phylogenetic trees were constructed using MEGA X [18] with the maximum–likelihood (ML) [19], neighbor–joining (NJ) [20], and maximum-parsimony (MP) [21] algorithms. The tree topologies were evaluated using the bootstrap method with 1,000 replicates [22]. Finally, evolutionary distances were calculated using Kimura’s two-parameter model [23].

### Genome Analysis

Genome sequencing for the W11^T^, SW19^T^, and YR1^T^ strains was performed using PacBio SMRT technology. Raw sequence data were assembled by NextDenovo v. 2.4.0. Genome sequence quality was assessed using the ContEst16S algorithm [24] and BLASTn tool [25]. Genome annotation was performed using the Prokaryotic Genome Annotation Pipeline (PGAP) [26] and Rapid Annotation Subsystem Technology (RAST) servers [27]. Functional annotation was performed using the eggNOG 4.5 database [28]. Orthologous gene cluster comparisons of the W11^T^, SW19^T^, and YR1^T^ strains with their closely related reference species were performed using the OrthoVenn3 tool [29]. The overall genome relatedness index (OGRI) of the W11^T^, SW19^T^, and YR1^T^ strains with their respective closest species was computed using digital DNA–DNA hybridization (dDDH) [30] and average nucleotide identity (ANI) [31] web-based tools. The gene cluster associated with potential secondary metabolites was assessed using antiSMASH 7.0. [32]. A phylogenomic tree was constructed on the Type (Strain) Genome Server with the FastME 2.1.6.1 tool [33].

### Morphological, Physiological, and Biochemical Analysis

The morphologies of the cells were visualized by transmission electron microscopy (Talos; FEI) after cultivating the W11^T^, SW19^T^, and YR1^T^ strains on R2A agar at 25°C for 5 days. Gram-staining was performed using the Color Gram 2 kit (bioMérieux, France). The anaerobic growth ability, motility test, and catalase and oxidase tests were performed as previously described [15]. The optimal growth parameters (temperature, NaCl, and pH ranges) were determined following previously described methods [34]. Tween 80, cellulose, DNA, casein, and starch hydrolysis were investigated according to standard protocols [35]. Other key enzymatic, carbon assimilation, and biochemical traits were assessed using API 20NE, API ID 32 GN, and API ZYM kits (bioMérieux).

### Chemotaxonomic Characterization

The W11^T^, SW19^T^, and YR1^T^ strains and their reference strains were cultivated at 25°C on R2A agar. Biomass was harvested in the late log phase and used for fatty acid analysis and identification following the MIDI protocol [36]. Peptidoglycan was investigated as previously reported [37]. Freeze-dried cells were prepared and used for the analysis of polar lipid and quinone levels using procedures outlined previously [38, 39]. Polar lipids were visualized by spraying with various reagents [40].

## Results and Discussion

**Fig. 1:**
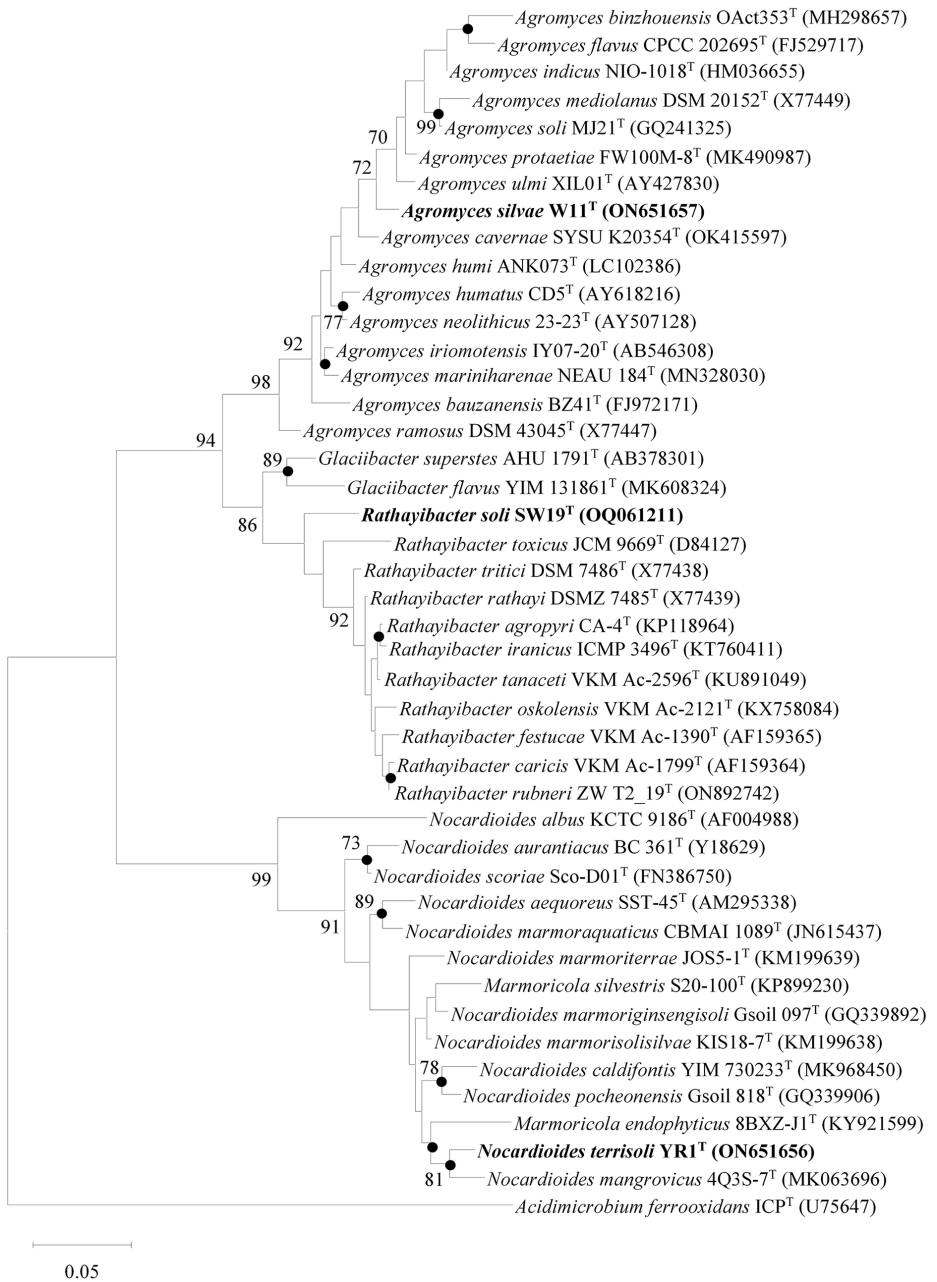
Maximum-likelihood tree generated based on the 16S rRNA gene sequences of strains W11^T^, SW19^T^, and YR1^T^. Branching nodes reproduced by both neighbor-joining and maximum-likelihood trees are denoted by filled circles. The numbers at the branching nodes indicate the percentage of 1,000 bootstrap replications (only values >70% are shown). GenBank accession numbers for 16S rRNA gene sequences are provided in parentheses. The scale bar corresponds to 0.05 substitutions per nucleotide position.

The lengths of the 16S rRNA gene sequences of the W11^T^, SW19^T^, and YR1^T^ strains were 1,457, 1,418, and 1,453 bp, respectively. Through 16S rRNA gene sequence analysis, we found that the W11^T^, SW19^T^, and YR1^T^ strains belonged to the genera *Agromyces*, *Rathayibacter*, and *Nocardioides*, respectively, and these strains exhibited the highest 16S rRNA gene sequence similarity to *Agromyces cavernae* SYSU K20354^T^ (98.1%), *Rathayibacter rubneri* ZW T2_19^T^ (97.0%), and *Nocardioides marmorisolisilvae* KIS18-7^T^ (98.0%), respectively. The 16S rRNA gene sequence identities of the strains studied with all other respective phylogenetically closest taxa were below the specified threshold values of < 98.7%, which is commonly used for species demarcation [41, 42]. Both ML and NJ trees delineated a distinct lineage for strain W11^T^ within the genus *Agromyces*, whereas strain SW19^T^ formed a separate lineage among the members of genus *Rathayibacter*. In contrast, strain YR1^T^ exhibited a monophyletic clade with *Nocardioides mangrovicus* 4Q3S-7^T^ in both phylogenetic trees (ML and NJ), suggesting that this strain may represent a novel member within the genus *Nocardioides* (Figs. 1 and S1).

The quality analysis confirmed that the genome sequences of the W11^T^, SW19^T^, and YR1^T^ strains were valid with no contamination. The genome sizes of strains W11^T^, SW19^T^, and YR1^T^ were 4,181,720, 4,740,677, and 4,228,226 bp, respectively, with the DNA G+C contents being 70.5%, 64.2%, and 69.7%, respectively. The genome sequences of all the three strains were assembled into a single contig with genome coverages of 121.0x for W11^T^, 119.0x for SW19^T^, and 124.0x for YR1^T^ (Table S1). Genome annotation of the W11^T^, SW19^T^, and YR1^T^ strains performed by RAST determined a total of 247, 258, and 268 subsystem features, respectively (Figs. S2, S3 and S4). The OGRI values based on the ANI and dDDH of the W11^T^, SW19^T^, and YR1^T^ strains with their closest taxa ranged between 72.1–79.6% and 19.6–23.6%, respectively (Table S2). These OGRI values were below the cut-off values of dDDH (70.0%) and ANI (95.0%), indicating the genomic distinctiveness of strains W11^T^, SW19^T^, and YR1^T^ from other phylogenetically affiliated taxa of the genera *Agromyces*, *Rathayibacter*, and *Nocardioides*, respectively [43, 44]. The phylogenomic tree constructed for W11^T^ showed the formation of a clade with *A. protaetiae* FW100M-8^T^ (Fig. S5). Strain SW19^T^ formed a separate lineage among the members of the genus *Rathayibacter* (Fig. S6). In addition, strain YR1^T^ formed a distinct lineage within the members of the genus *Nocardioides* in the phylogenomic tree (Fig. S7).

**Fig. 2:**
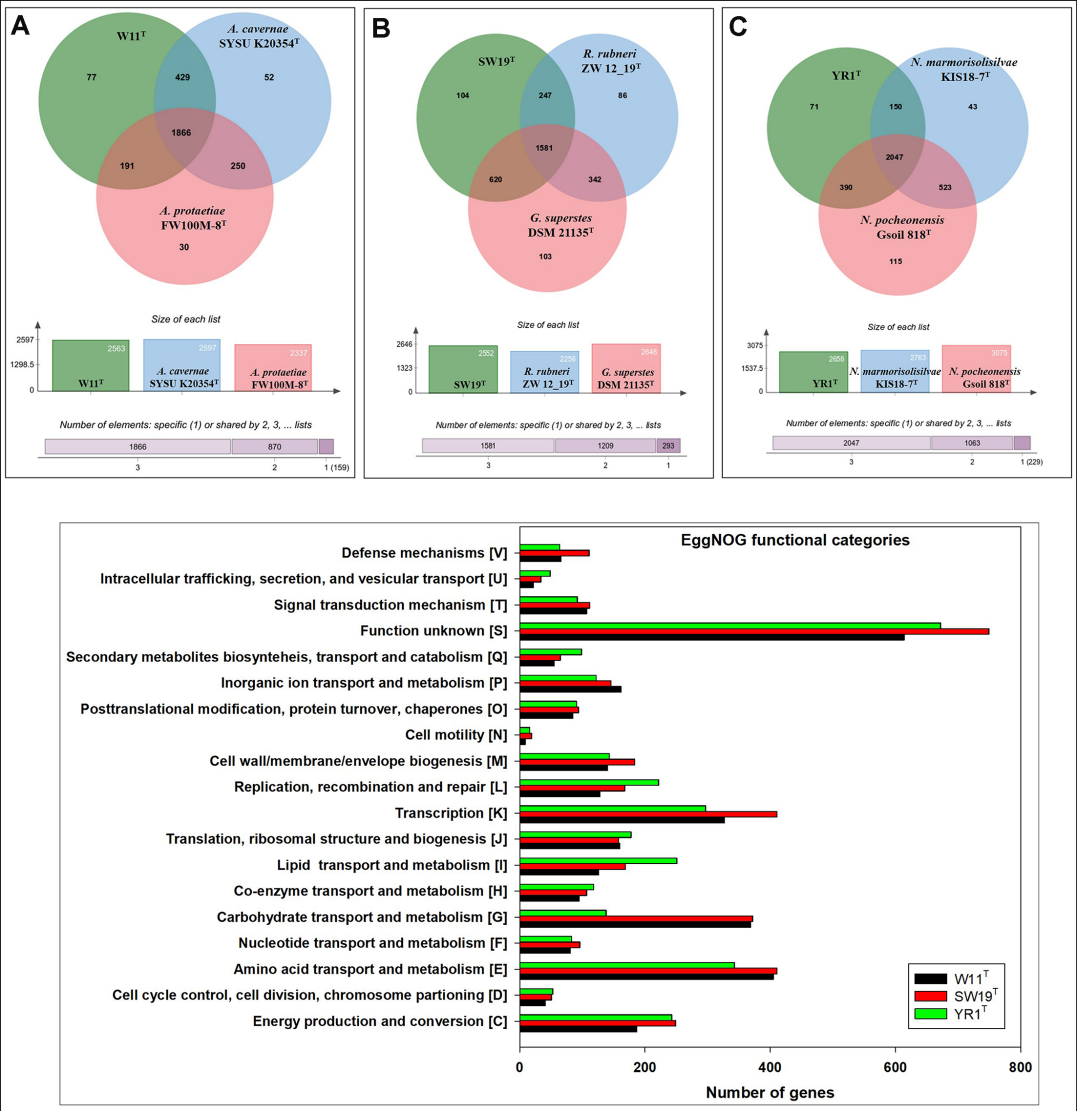
Distributions of gene clusters into nineteen EggNOG functional categories of strains W11^T^, SW19^T^, and YR1^T^, along with Venn diagrams depicting orthologous genes of each strain (A, W11^T^; B, SW19^T^; and C, YR1^T^).

The orthologous gene clusters of strains W11^T^, SW19^T^, and YR1^T^ extracted from the genome dataset were analyzed through the EggNOG pipeline. A total of 3,179, 3,706, and 3,274 genes, respectively, were categorized into 19 functional groups. Most genes were clustered into unknown functional category [S], accounting for a total of 614, 749, and 672 genes, respectively, for the W11^T^, SW19^T^, and YR1^T^ strains. In known functional categories, most genes of the W11^T^, SW19^T^, and YR1^T^ strains were related to amino acid transport and metabolism [E], carbohydrate transport and metabolism [G], transcription [K], lipid transport and metabolism [I], and energy production and conversion [C]. The EggNOG functional analysis of all three strains indicated that the cell motility [N] category accounted the lowest number of genes (Fig. 2). The Venn diagrams in Fig. 2A, 2B, and 2C indicate the comparative genomic data for the W11^T^, SW19^T^, and YR1^T^ strains, respectively. Fig. 2A shows that strains W11^T^, *Agromyces cavernae* SYSU K20354^T^, and *Agromyces protaetiae* FW100M-8^T^ shared 1,866 orthologous genes, while only 77 genes were unique to strain W11^T^. Similarly, Fig. 2B shows that strains SW19^T^, *Rathayibacter rubneri* ZW 12_19^T^, and *Glaciibacter superstes* DSM 21135^T^ shared 1,581 orthologous genes, with 104 genes being unique to strain SW19^T^. Fig. 2C depicts that strains YR1^T^, *Nocardioides marmorisolisilvae* KIS18-7^T^, and *Nocardioides pocheonensis* Gsoil 818^T^ shared 2,047 identical genes, whereas 71 genes were unique to strain YR1^T^. Clusters encoding for terpene were observed in the genomes of all three strains through biosynthetic gene cluster analysis. The genes encoding betalactone and butyrolactone were detected in strains W11^T^ and SW19^T^. Gene clusters for Type II polyketide synthase were exclusively observed in strain W11^T^, whereas gene clusters synthesizing non-alpha poly-amino acids such as e-polylysin and proteusin were found only in the genome of strain SW19^T^ (Table S3).

All three strains exhibited Gram positive and rod-shaped morphologies (Figs. S8, S9, and S10). The temperature ranges for the growth of the W11^T^, SW19^T^, and YR1^T^ strains were 18–37°C, 10–30°C, and 10–37°C, respectively. Strain W11^T^ thrived in up to 4.0% NaCl, whereas strains SW19^T^ and YR1^T^ could tolerate only up to 1.0% NaCl. The pH ranges for the growth of the W11^T^, SW19^T^, and YR1^T^ strains were 6.5–9.0, 6.5–9.5, and 5.5–8.0, respectively. Strains W11^T^ and SW19^T^ could perform DNA hydrolysis, whereas strain YR1^T^ lacked this ability. Other major distinguishing features of strains W11^T^, SW19^T^, and YR1^T^ are presented in the species protologue and illustrated in Table 1 along with the phylogenetically closest reference taxa. A complete set of enzymatic and carbon assimilative data obtained from API kits is provided in Table S4.

In strain W11^T^, MK-11 (52.8%) and MK-12 (47.2%) were found to be the predominant menaquinones; this result agrees with the findings reported for other members of the genus *Agromyces* [5, 6]. Similarly, in strain SW19^T^, MK-12 (65.4%), MK-11 (19.5%), and MK-13 (15.1%) were found to be the primary menaquinones, which is consistent with observations in other species of the genus *Rathayibacter* [2, 13]. In contrast, for strain YR1^T^, we detected that MK-8(H_4_) was the exclusive menaquinone, in line with observations in other members of the genus *Nocardioides* [3, 12]. The predominant polar lipids observed in strains W11^T^ and SW19^T^ were diphosphatidylglycerol, phosphatidylglycerol, and an unidentified glycolipid. In contrast, the primary polar lipids in strain YR1^T^ were phosphatidylglycerol, phosphatidylinositol, and phosphatidylcholine (Fig. S11). The polar lipid profiles of strains W11^T^, SW19^T^, and YR1^T^ resemble their respective closely related members of the genera *Agromyces*, *Rathayibacter*, and *Nocardioides* [6, 11, 13]. The peptidoglycans in strains W11^T^ and SW19^T^ included 2,4-diaminobutyric acid, alanine, glycine, and glutamic acid, whereas strain YR1^T^ contained LL-diaminopimelic acid as the diagnostic peptidoglycan. The main cellular fatty acids identified in strain W11^T^ were anteiso-C_15:0_ (41.2%), iso-C_16:0_ (24.3%), and anteiso-C_17:0_ (23.9%). In strain SW19^T^, the main cellular fatty acids were feature 9 (C_16:0_ 10-methyl and/or iso-C_17:1_w 9c; 50.8%), anteiso-C_17:0_ (14.7%), and anteiso-C_15:0_ (13.0%). The primary cellular fatty acids in strain YR1^T^ were C_18:1_w 9c (25.1%), C_18:0_ 10-methyl, TBSA (23.2%), and anteiso-C_15:0_ (11.4%). While the major fatty acids of strains W11^T^, SW19^T^, and YR1^T^ were consistent with those of the reference species, there were proportional differences in the composition of minor fatty acids among these strains and their closest taxa, as shown in Table 2.

### Taxonomic Conclusion

Through comprehensive taxonomic analysis, we showed that strains W11^T^, SW19^T^, and YR1^T^ belong to new species within the genera *Agromyces*, *Rathayibacter*, and *Nocardioides*, respectively. The proposed names of the strains W11^T^, SW19^T^, and YR1^T^ are *Agromyces silvae* sp., *Rathayibacter soli* sp. nov., and *Nocardioides terrisoli*sp. nov., respectively.

### Description of *Agromyces silvae* sp. nov.

*Agromyces silvae* sp. nov. (sil'vae. L. gen. n. *silvae* of a forest).

Cells (5.2–5.9 × 1.7–1.9 μm) are strictly aerobic, Gram-stain-positive, non-motile, and rod-shaped. Colonies are yellow-coloured, smooth, convex, and circular with the diameter of 4.8–5.6 mm. Cells grow at temperature of 18–37°C (optimum 25-28°C), at pH of 6.5–9.0 (optimum 7.5), and at NaCl content of 0–4.0% (w/v) (optimum without NaCl). Negative for catalase, oxidase, and nitrate reduction. Hydrolyse DNA and esculin, but cannot hydrolyse starch, casein, cellulose Tween 80, gelatin, and urea. Positive for esterase (C4), leucine arylamidase, acid phosphatase, naphthol-AS-BI-phosphohydrolase, *β*-galactosidase, and *α*-glucosidase. Weakly positive for valine arylamidase and cystine arylamidase. Assimilates D-glucose, D-mannitol, D-maltose, gluconate, L-rhamnose, D-ribose, inositol, D-sucrose, acetate, and lactate. The predominant menaquinones are MK-11 and MK-12; key cellular fatty acids are anteiso-C_15:0_, iso-C_16:0_, and anteiso-C_17:0_; main peptidoglycans are 2,4-diaminobutyric acid, alanine, glycine, and glutamic acid; and major polar lipids are diphosphatidylglycerol, phosphatidylglycerol, and an unidentified glycolipid. The DNA G+C content is 70.5%.

The type strain, W11^T^ (=KCTC 49818^T^ =NBRC 115999^T^), was isolated from soil in Republic of Korea (36°47'30.1"N 127°29'47.0"E).

The GenBank/EMBL/DDBJ accession numbers for the 16S rRNA nucleotide and genome sequences of strain W11^T^ are ON651657 and CP133018, respectively.

### Description of *Rathayibacter soli* sp. nov.

*Rathayibacter soli* sp. nov. (so’li. L. gen. n. soli of the soil).

Cells (1.2–1.4 × 0.5–0.7 μm) are strictly aerobic, Gram-stain-positive, non-motile, and rod-shaped. Colonies are white-coloured, smooth, convex, and circular with the diameter of 1.1–1.5 mm. Cells grow at temperature of 10–30°C (optimum 25°C), at pH of 6.5–9.5 (optimum 7.0), and at NaCl content of 0–1.0% (w/v) (optimum without NaCl). Positive for catalase, and negative for oxidase and nitrate reduction. Hydrolyse DNA and esculin, but cannot hydrolyse starch, casein, cellulose Tween 80, gelatin, and urea. Positive for esterase (C4), esterase lipase (C8), leucine arylamidase, acid phosphatase, naphthol-AS-BI-phosphohydrolase, *α*-galactosidase, *β*-galactosidase, *α*-glucosidase, *β*-glucosidase, *N*–acetyl-*β*-glucosaminidase, and *α*-mannosidase. Assimilates D-glucose, D-mannose, D-melibiose, and D-ribose. The predominant menaquinones are MK-12, MK-11, and MK-13; key cellular fatty acids are summed feature 9 (C_16:0_ 10-methyl and/or iso-C_17:1_*ω* 9c), anteiso-C_17:0_, and anteiso-C_15:0_; main peptidoglycans are 2,4-diaminobutyric acid, alanine, glycine, and glutamic acid; and major polar lipids are diphosphatidylglycerol, phosphatidylglycerol, and an unidentified glycolipid. The DNA G+C content is 64.2%.

The type strain, SW19^T^ (=KCTC 49860^T^ =NBRC 116108^T^), was isolated from soil in Republic of Korea (37°05'57.1"N 127°02'19.0"E).

The GenBank/EMBL/DDBJ accession numbers for the 16S rRNA nucleotide and genome sequences of strain SW19^T^ are OQ061211 and CP133020, respectively.

### Description of *Nocardioides terrisoli*sp. nov.

*Nocardioides terrisoli*sp. nov. (ter.ri.sóli. L. fem. n. *terra*, the earth; L. neut. n. *solum*, soil; N.L. gen. n. *terrisoli*, of soil of the earth).

Cells (0.9–1.0 × 0.5–0.6 μm) are strictly aerobic, Gram-stain-positive, non-motile, and rod-shaped. Colonies are yellow-coloured, smooth, convex, and circular with the diameter of 1.2–1.7 mm. Cells grow at temperature of 10–37°C (optimum 25°C), at pH of 5.5–8.0 (optimum 7.0), and at NaCl content of 0–1.0% (w/v) (optimum without NaCl). Positive for catalase, oxidase, and nitrate reduction. DNA, esculin, starch, casein, cellulose Tween 80, gelatin, and urea are not hydrolysed. Positive for esterase (C4), esterase lipase (C8), leucine arylamidase, acid phosphatase, naphthol-AS-BI-phosphohydrolase, and *α*-glucosidase. Assimilates propionate, valerate, and L-histidine. The sole menaquinone is MK-8(H_4_); key cellular fatty acids are C_18:1_*w9c*, C_18:0_ 10-methyl, TBSA, and anteiso-C_15:0_; diagnostic peptidoglycan is LL-diaminopimelic acid; and major polar lipids are phosphatidylglycerol, phosphatidylinositol, and phosphatidylcholine. The DNA G+C content is 69.7%.

The type strain, YR1^T^ (=KCTC 49863^T^ =NBRC 116165^T^), was isolated from soil in Republic of Korea (36°30'20.2"N 127°01'13.1"E).

The GenBank/EMBL/DDBJ accession numbers for the 16S rRNA nucleotide and genome sequences of strain YR1^T^ are ON651656 and CP134227, respectively.

## Supplemental Materials

Supplementary data for this paper are available on-line only at http://jmb.or.kr.



## Figures and Tables

**Table 1:** Differentiating properties of strains W11^T^, SW19^T^, and YR1^T^, and related reference members.

Characteristic	1	2	3	4	5	6	7	8	9	10	11	12	13
Growth temperature (°C)	18–37	4–37	15–35	10–35	15–37	10–30	10–35	-5–25	10–37	10–35	10–37	10–35	10–35
Highest salt tolerance (%, w/v)	4.0	4.0	3.0	3.0	3.0	1.0	6.0	4.0	1.0	1.0	1.0	3.0	10.0
pH range	6.5–9.0	6.0–8.0	6.5–9.0	6.0–8.5	6.0–8.0	6.5–9.5	6.5–9.0	5.5–11.0	5.5–8.0	6.0–8.0	6.0–8.0	5.0–8.0	6.0–8.0
Catalase/oxidase	-/-	+/-	-/-	+/-	-/-	+/-	+/+	+/-	+/+	+/+	+/+	+/-	+/-
Motility	-	-	-	-	-	-	-	+	-	+	-	+	-
**Hydrolysis of**													
Starch	-	-	+	-	-	-	-	-	-	-	-	+	+
Casein	-	-	-	-	+	-	-	-	-	+	+	+	+
DNase	+	-	-	-	-	+	-	-	-	-	-	-	-
Esculin	+	+	+	+	+	+	-	+	-	+	+	+	-
Gelatin	-	-	-	-	-	-	-	-	-	+	-	+	+
**Enzyme activity**													
Alkaline	-	-	+	-	-	+	-	-					
phosphatase													
Esterase Lipase (C8)	-	-	-	-	-	-	+	-	-	-	-	+	+
Leucine arylamidase	-	+	+	+	+	+	+	+	+	+	+	+	+
Valine arylamidase	+	+	+	+	+	+	+	+	+	w	-	+	+
Cystine arylamidase	w	+	w	+	w	-	+	+	-	-	-	-	+
*α*-Galactosidase	w	+	w	+	w	-	-	w	-	-	-	-	+
*β*-Galactosidase	-	-	-	-	-	+	+	+	-	-	-	-	-
*α*-Glucosidase	+	+	-	+	-	+	+	+	-	-	+	-	-
*β*-Glucosidase	+	+	+	+	+	+	+	+	+	-	+	+	+
*N*–Acetyl -*β*-glucosaminidase	-	+	+	+	+	+	+	+	-	+	-	-	-
**Assimilation from**													
(API 20NE and ID 32 GN test)													
D-Glucose	+	-	+	+	+	+	+	+	-	-	+	+	+
L-Arabinose	-	-	-	-	-	-	-	+	-	-	+	-	-
D-Mannose	-	-	+	+	+	+	-	+	-	-	-	+	-
D-Mannitol	+	-	+	-	+	-	+	+	-	-	-	+	+
D-Maltose	+	-	+	+	+	-	-	+	-	-	-	-	-
Gluconate	+	-	-	-	-	-	-	+	-	-	-	-	-
Malate	-	-	-	+	-	-	-	-	-	-	-	-	+
D-Melibiose	-	-	-	-	-	+	-	-	-	-	+	-	-
Propionate	-	-	-	-	-	-	-	-	+	-	-	-	w
Valerate	-	-	-	-	-	-	-	-	+	-	+	-	+
L-Histidine	-	-	-	+	-	-	-	-	+	-	-	-	-
D-Rhamnose	+	-	-	+	+	-	-	+	-	-	-	-	-
D-Ribose	+	-	-	+	-	+	-	+	-	-	-	+	-
Inositol	+	+	-	-	-	-	-	-	-	-	+	+	-
D-Sucrose	+	+	+	+	+	-	+	-	-	-	-	-	+
Acetate	+	-	-	+	-	-	-	-	-	-	-	-	-
Lactate	+	-	+	+	-	-	-	-	-	-	-	-	-
DNA G + C content (%)	70.5	69.8	70.5	72.4	72.0	64.2	71.8	64.4	69.7	69.6	71.0	72.0	72.3

Strains: 1, W11^T^; 2, *Agromyces cavernae* KCTC 49499^T^; 3, *Agromyces protaetiae* KACC 19308^T^; 4, *Agromyces mediolanus* KCTC 3136^T^; 5, *Agromyces ulimi* KACC 20592^T^; 6, SW19^T^; 7, *Rathayibacter rubneri* DSM 114294^T^ ; 8, *Glaciibacter superstes* NBRC 104264^T^ ; 9, YR1^T^ ; 10, *Nocardioides marmorisolisilvae* KACC 17307^T^; 11, *Nocardioides pocheonensis* KACC 14275^T^ ; 12, *Nocardioides mangrovicus* KCTC 39790^T^; 13, *Marmoricola endophyticus* KCTC 39790^T^. The G+C content data were computed from genome sequence of respective strains. +, positive; w, weakly positive; -, negative.

**Table 2:** Cellular fatty acid profiles (% of totals) of strains W11^T^, SW19^T^, YR1^T^, and phylogenetically related reference species.

Fatty acid	1	2	3	4	5	6	7	8	9	10	11	12	13
**Saturated**													
C_10:0_	–	–	–	tr	–	tr	–	–	1.9	–	–	–	–
C_12:0_	–	–	–	1.0	tr	tr	–	–	1.9	tr	tr	tr	–
C_14:0_	–	–	tr	tr	tr	tr	–	tr	tr	1.1	tr	tr	tr
C_16:0_	1.5	2.3	3.0	2.2	1.0	tr	1.1	tr	**9.5**	5.7	1.2	1.5	6.9
C_17:0_	–	–	–	–	–	–	–	tr	–	3.9	1.0	2.5	4.3
C_18:0_	–	–	tr	tr	–	tr	–	tr	3.9	1.8	0.8	6.9	6.0
**Unsaturated**													
C_17:1_ ω8c	–	–	–	–	–	–	–	–	1.1	6.4	2.4	3.7	1.6
C_18:1_ ω9c	–	–	tr	tr	–	–	TR	–	**25.1**	6.3	3.2	15.6	4.5
Branched saturated													
iso-C_13:0_	–	–	–	–	–	–	tr	–	–	1.1	TR	–	–
iso-C_14:0_	1.6	1.9	1.1	2.0	2.4	tr	–	tr	–	9.6	5.6	tr	tr
iso-C_15:0_	5.7	6.8	5.5	6.8	4.1	2.7	4.8	6.0	2.4	10.4	10.6	1.0	2.2
iso-C_16:0_	**24.3**	34.9	23.3	29.7	28.6	**10.4**	17.8	7.2	**11.4**	28.0	48.3	23.7	18.4
iso-C_16:1_ H	–	–	–	–	–	–	–	–	1.0	1.7	5.1	–	–
iso-C_17:0_	1.4	2.2	1.7	1.7	1.0	4.4	2.9	1.6	2.4	3.8	5.5	4.5	1.3
iso-C_18:0_	–	–	–	–	–	tr	–	–	tr	1.0	1.0	2.8	1.4
anteiso-C_15:0_	**41.2**	31.2	38.6	36.6	42.3	**13.0**	49.6	51.3	–	1.7	1.4	–	tr
anteiso-C_15:1_ A	tr	–	tr	tr	tr	–	–	9.6	–	–	–	–	–
anteiso-C_17:0_	**23.9**	19.5	24.2	18.0	19.6	**14.7**	16.9	20.8	1.1	1.5	2.0	2.0	1.0
C_17:0_ 10-methyl	–	–	–	–	–	–	–	–	–	2.9	–	–	7.1
C_18:0_ 10-methyl, TBSA	–	–	–	–	–	–	–	–	**23.2**	1.2	2.1	21.0	29.0
**Hydroxy fatty acid**													
C_15:0_ 2OH	–	–	–	–	–	–	–	–	1.8	tr	–	–	tr
C_16:0_ 2OH	–	–	–	–	–	–	–	–	–	tr	–	1.0	5.0
C_17:0_ 2OH	–	–	–	–	–	–	–	–	–	–	–	1.1	2.3
C_17:0_ 3OH	–	–	–	–	–	–	–	–	–	–	–	1.0	1.3
**Summed features** *													
3	–	–	–	–	–	tr	–	–	**8.5**	4.6	1.2	tr	1.3
6	–	–	–	–	–	–	–	–	–	–	tr	2.9	tr
8	–	–	–	–	–	**50.8**	5.6	3.2	5.3	1.7	1.0	5.2	1.4
9	–	–	–	–	–	–	–	–	–	2.2	4.7	1.0	1.8

Strains: 1, W11^T^; 2, *Agromyces cavernae* KCTC 49499^T^; 3, *Agromyces protaetiae* KACC 19308^T^; 4, *Agromyces mediolanus* KCTC 3136^T^; 5, *Agromyces ulimi* KACC 20592^T^; 6, SW19^T^; 7, *Rathayibacter rubneri* DSM 114294^T^ ; 8, *Glaciibacter superstes* NBRC 104264^T^ ; 9, YR1^T^ ; 10, *Nocardioides marmorisolisilvae* KACC 17307^T^; 11, *Nocardioides pocheonensis* KACC 14275^T^ ; 12, *Nocardioides mangrovicus* KCTC 39790^T^; 13, *Marmoricola endophyticus* KCTC 39790^T^. All the data were from this study. TR, trace amount (<1.0%); –, not detected.

*Summed features represent groups of two or three fatty acids that could not be separated using the MIDI system. Summed feature 3 comprised C_16:1_*ω*7c and/or C_16:1_*ω* 6c; Summed feature 6 comprised C_19:1_*ω*11c and/or C_19:1_*ω*9c; Summed feature 8 comprised C_18:1_*ω*7c and/or C_18:1_*ω*6c; and Summed feature 9 comprised C_16:0_ 10-methyl and/or iso-C17: 1*ω*9c. Unknown fatty acids are designated by their ECL, relative to the chain lengths of known straight-chain saturated fatty acids.
