# Supplementary material for: Agromyces silvae sp. nov., Rathayibacter soli sp. nov., and Nocardioides terrisolisp. nov., Isolated from Soil
Source: J Microbiol Biotechnol. 2024 May 23;34(7):1475–83. doi: 10.4014/jmb.2404.04007 (PMC11294650; doi:10.4014/jmb.2404.04007)
Supplement: Supplementary file 1 [file jmb-34-7-1475-supple.pdf]

J. Microbiol. Biotechnol. 2024. 34(7): 1475–1483

https://doi.org/10.4014/jmb.2404.04007

Supplementary Tables and Figures

Agromyces silvae sp. nov., *Rathayibacter soli* sp. nov., and *Nocardioides terrisoli* sp. nov., Isolated from Soil

Hyosun Lee [1], Dhiraj Kumar Chaudhary [2], and Dong-Uk Kim [1*]

[1] Department of Biological Science, College of Science and Engineering, Sangji University, Wonju, 26339, Republic of Korea

[2] Department of Microbiology, Pukyong National University, Busan, 48513, Republic of Korea

*Dong-Uk Kim (dukim@sangji.ac.kr)

Supplementary Table S1. Genome feature of strains W11^{T}, SW19^{T} and YR1^{T}.

**Genome features**	**W11^{T}**	**SW19^{T}**	**YR1^{T}**
Genome size (bp)	4,181,720	4,740,677	4,228,226
G + C content (%)	70.5	64.2	69.7
No. of contigs	1	1	1
N50 (bp)	4,181,720	4,740,677	4,228,226
L50	1	1	1
No. of subsystem	247	258	268
No. of proteins	3,745	4,313	3,954
Total genes	3,817	4,420	4,038
CDSs (total)	3,761	4,364	3,986
Protein-coding genes	3,745	4,313	3,954
Genes (RNA)	56	56	52
rRNAs (5S, 16S, 23S)	2, 2, 2	2, 2, 2	1, 1, 1
Complete rRNAs (5S)	2, 2, 2	2, 2, 2	1, 1, 1
tRNAs	46	47	46
ncRNAs	4	3	3
Pseudo Genes (total)	16	51	32
Genome coverage	121.0x	119.0x	124.0x

Table S2. Average nucleotide identity (ANI) and digital DNA-DNA hybridization (dDDH) values between strains W11^{T}, SW19^{T}, YR1^{T} and phylogenetically closest members.

Reference strains	W11^{T}	
	**ANI (%)**	**dDDH (%)**
Agromyces cavernae SYSU K20354^{T}	78.8	22.3
Agromyces protaetiae FW100M-8^{T}	79.5	22.8
Agromyces mediolanus JCM 3346^{T}	78.9	22.3
Agromyces mariniharenae NEAU-184^{T}	79.6	23.1
Agromyces humi ANK073^{T}	79.5	23.6
Agromyces flavus CPCC 202695^{T}	78.6	22.1
Agromyces bauzanensis CGMCC 1.8984^{T}	78.7	22.3
Agromyces neolithicus JCM 14322^{T}	78.7	22.4
Agromyces binzhouensis CGMCC 4.7180^{T}	79.1	23.0
Agromyces indicus CCTCC AB 2011122^{T}	78.6	22.1
**Reference strains**	**SW19^{T}**	
	**ANI (%)**	**dDDH (%)**
Rathayibacter rubneri ZW T2_19^{T}	72.3	20.6
Rathayibacter tritici DSM 7486^{T}	72.2	21.3
Glaciibacter superstes DSM 21135^{T}	72.1	20.4
Glaciibacter flavus YIM 131861^{T}	72.2	19.7
**Reference strains**	**YR1^{T}**	
	**ANI (%)**	**dDDH (%)**
Nocardioides marmorisolisilvae KIS18-7^{T}	75.7	19.7
Nocardioides pocheonensis Gsoil 818^{T}	76.4	20.4
Nocardioides mangrovicus 4Q3S-7^{T}	75.3	19.8
Nocardioides marmoriginsengisoli Gsoil 097^{T}	75.9	19.8
Nocardioides aequoreus NRRL B-24464^{T}	75.7	20.2
Nocardioides caldifontis YIM 730233^{T}	74.5	19.6

Table S3. Distribution of biosynthetic gene clusters (BGCs) in the genome of the strains W11^{T}, SW19^{T}, and YR1^{T}.

**W11^{T}**					
**Genomic regions**	**Type**	**From**	**To**	**Most similar known cluster**	**Similarity (%)**
Region 1	Terpene	328,690	349,571	Carotenoid	50
Region 2	Butyrolactone	688,683	699,477		
Region 3	Betalactone	2,389,780	2,415,761	Microansamycin	7
Region 4	T3PKS	3,694,002	3,735,144		
**SW19^{T}**					
Region 1	T3PKS	78,910	120,214	Alkylresorcinol	100
Region 2	Betalactone	944,602	977,127		
Region 3	Betalactone	2,144,555	2,171,757	Microansamycin	7
Region 4	Terpene	2,877,981	2,898,877	Carotenoid	21
Region 5	Butyrolactone	2,920,026	2,930,871		
Region 6	NAPAA	4,156,438	4,190,463	ε-Poly-L-lysine	100
Region 7	Proteusin, LAP	4,511,795	4,542,123		
**YR1^{T}**					
Region 1	Terpene	1,722,013	1,748,089	Hopene	53

T3PKS: Type III Polyketide synthase; NAPAA: Non-alpha poly-amino acids like e-Polylysin; LAP: Linear azol(in)e-containing peptides.

Table S4. The enzymatic and assimilation data obtained from API ZYM, API 20NE, and API ID 32 GN tests of strains W11^{T}, SW19^{T}, YR1^{T}, and related reference members. Strains: 1, W11^{T}; 2, *Agromyces cavernae* KCTC 49499^{T}; 3, *Agromyces protaetiae* KACC 19308^{T}; 4, *Agromyces mediolanus* KCTC 3136^{T}; 5, *Agromyces ulimi* KACC 20592^{T}; 6, SW19^{T}; 7, *Rathayibacter rubneri* DSM 114294^{T}; 8, *Glaciibacter superstes* NBRC 104264^{T}; 9, YR1^{T}; 10, *Nocardioides marmorisolisilvae* KACC 17307^{T}; 11, *Nocardioides pocheonensis* KACC 14275^{T}; 12, *Nocardioides mangrovicus* KCTC 39790^{T}; 13, *Marmoricola endophyticus* KCTC 39790^{T}. +, positive; w, weakly positive; -, negative.

**API ZYM test**	**1**	**2**	**3**	**4**	**5**	**6**	**7**	**8**	**9**	**10**	**11**	**12**	**13**
Alkaline phosphatase	-	-	-	-	-	-	+	-	-	-	-	+	+
Esterase (C4)	+	+	+	+	+	+	+	+	+	+	+	+	+
Esterase Lipase (C8)	-	+	+	+	+	+	+	+	+	+	+	+	+
Lipase (C14)	-	-	-	-	-	-	-	-	-	-	-	-	-
Leucine arylamidase	+	+	+	+	+	+	+	+	+	w	-	+	+
Valine arylamidase	w	+	w	+	w	-	+	+	-	-	-	-	+
Cystine arylamidase	w	+	w	+	w	-	-	w	-	-	-	-	+
Trypsin	-	+	-	-	-	-	-	w	-	-	-	-	-
α-Chymotrypsin	-	-	-	-	-	-	-	-	-	-	-	-	-
Acid phosphatase	+	-	+	+	+	+	+	+	+	+	+	+	+
Naphthol-AS-BI-phosphohydrolase	+	+	+	+	+	+	+	+	+	+	+	+	+
α-Galactosidase	-	-	-	-	-	+	+	+	-	-	-	-	-
β-Galactosidase	+	+	-	+	-	+	+	+	-	-	+	-	-
β-Glucuronidase	-	-	-	-	-	-	-	-	-	-	-	-	-
α-Glucosidase	+	+	+	+	+	+	+	+	+	-	+	+	+
β-Glucosidase	-	+	+	+	+	+	+	+	-	+	-	-	-
N–Acetyl -*β*-glucosaminidase	-	-	+	+	+	+	-	-	-	-	-	-	-
α-Mannosidase	-	-	-	-	-	+	-	+	-	-	-	-	-
α-Fucosidase	-	-	-	-	-	-	-	-	-	-	-	-	-
**API 20NE test**													
Reduction of nitrates (NO_3^-) to nitrites (NO_2^-)	-	-	-	-	-	-	-	-	+	-	+	-	-

Reduction of nitrates (NO_3^-) to nitrogen(N_2)	-	-	-	-	-	-	-	-	-	-	-	-	-
Indole production	-	-	-	-	-	-	-	-	-	-	-	-	-
Glucose Acidification	-	-	-	-	-	-	-	-	-	-	-	-	-
Arginine dihydrolase	-	-	-	-	-	-	-	-	-	-	-	-	-
Urease	-	-	-	-	-	-	-	-	-	-	-	-	-
β-Glucosidase (esculin hydrolysis)	+	+	+	+	+	+	-	+	-	+	+	+	-
Protease (gelatin hydrolysis)	-	-	-	-	-	-	-	-	-	+	-	+	+
β-Galactosidase (PNPG)	+	+	-	+	-	-	-	+	-	-	+	-	-
D-Glucose	+	-	+	+	+	+	+	+	-	-	+	+	+
L-Arabinose	-	-	-	-	-	-	-	+	-	-	+	-	-
D-Mannose	-	-	+	+	+	+	-	+	-	-	-	+	-
D-Mannitol	+	-	+	-	+	-	+	+	-	-	-	+	+
N-Acetyl- D-glucosamine	-	-	-	+	-	-	-	-	-	-	-	-	-
D-Maltose	+	-	+	+	+	-	-	+	-	-	-	-	-
Gluconate	+	-	-	-	-	-	-	+	-	-	-	-	-
Caprate	-	-	-	-	-	-	-	-	-	-	-	-	-
Adipate	-	-	-	-	-	-	-	-	-	-	-	-	-
Malate	-	-	-	+	-	-	-	-	-	-	-	-	+
Citrate	-	-	-	-	-	-	-	-	-	-	-	-	-
Phenyl-acetate	-	-	-	+	-	-	-	-	-	-	-	-	-
**API ID 32 GN test**													
D-Mannitol	+	-	+	-	+	-	+	+	-	-	-	+	+
D-Glucose	+	-	+	+	+	+	+	+	-	-	+	+	+
Salicin	-	-	-	+	-	-	-	-	-	-	-	-	-
D-Melibiose	-	-	-	-	-	+	-	-	-	-	+	-	-
L-Fucose	-	-	+	-	-	-	-	-	-	-	-	-	-
D-Sorbitol	-	-	-	-	-	-	-	-	-	-	-	-	-

L-Arabinose	-	-	-	-	-	-	-	+	-	-	+	-	-
Propionate	-	-	-	-	-	-	-	-	+	-	-	-	w
Caprate	-	-	-	-	-	-	-	-	-	-	-	-	-
Valerate	-	-	-	-	-	-	-	-	+	-	+	-	+
Citrate	-	-	-	-	-	-	-	-	-	-	-	-	-
L-Histidine	-	-	-	+	-	-	-	-	+	-	-	-	-
2-Ketogluconate	-	-	-	-	-	-	-	-	-	-	-	-	-
3-Hydroxy-butyrate	-	-	-	-	-	-	-	-	-	-	-	-	+
4-Hydroxy-benzoate	-	-	-	-	-	-	-	-	-	-	-	-	-
L-Proline	-	-	-	-	-	-	-	-	-	-	-	-	-
L-Rhamnose	+	-	-	+	+	-	-	+	-	-	-	-	-
N-Acetyl- D-glucosamine	-	-	-	+	-	-	-	-	-	-	-	-	-
D-Ribose	+	-	-	+	-	+	-	+	-	-	-	+	-
Inositol	+	+	-	-	-	-	-	-	-	-	+	+	-
D-Sucrose	+	+	+	+	+	-	+	-	-	-	-	-	+
D-Maltose	+	-	+	+	+	-	-	+	-	-	-	-	-
Itaconate	-	-	-	-	-	-	-	-	-	-	-	-	-
Suberate	-	-	-	-	-	-	-	-	-	-	-	-	+
Malonate	-	-	-	-	-	-	-	-	-	-	-	-	-
Acetate	+	-	-	+	-	-	-	-	-	-	-	-	-
Lactate	+	-	+	+	-	-	-	-	-	-	-	-	-
L-Alanine	-	+	-	+	-	-	-	-	-	-	-	-	-
5-Ketogluconate	-	-	-	-	-	-	-	-	-	-	-	-	-
Glycogen	-	-	+	-	-	-	-	-	-	-	-	-	-
3-Hydroxy-benzoate	-	-	-	-	-	-	-	-	-	-	-	-	-
L-Serine	-	+	-	-	-	-	-	-	-	-	-	-	-

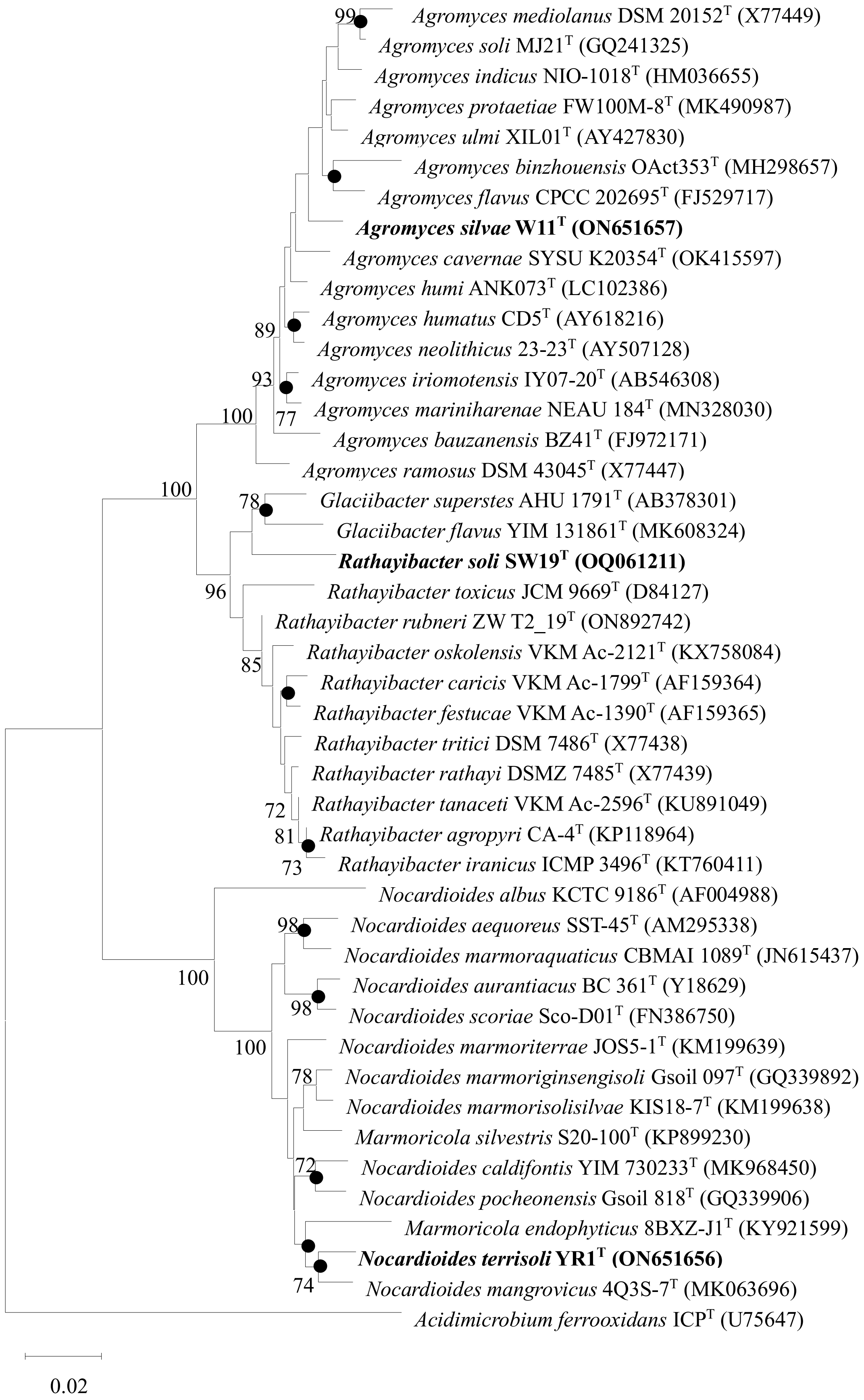
Agromyces mediolanus DSM 20152^{T} (X77449)
Agromyces soli MJ21^{T} (GQ241325)
Agromyces indicus NIO-1018^{T} (HM036655)
Agromyces protaetiae FW100M-8^{T} (MK490987)
Agromyces ulmi XIL01^{T} (AY427830)
Agromyces binzhouensis OAct353^{T} (MH298657)
Agromyces flavus CPCC 202695^{T} (FJ529717)
***Agromyces silvae* W11^{T} (ON651657)**
Agromyces cavernae SYSU K20354^{T} (OK415597)
Agromyces humi ANK073^{T} (LC102386)
Agromyces humatus CD5^{T} (AY618216)
Agromyces neolithicus 23-23^{T} (AY507128)
Agromyces iriomotensis IY07-20^{T} (AB546308)
Agromyces mariniharenae NEAU 184^{T} (MN328030)
Agromyces bauzanensis BZ41^{T} (FJ972171)
Agromyces ramosus DSM 43045^{T} (X77447)
Glaciibacter superstes AHU 1791^{T} (AB378301)
Glaciibacter flavus YIM 131861^{T} (MK608324)
***Rathayibacter soli* SW19^{T} (OQ061211)**
Rathayibacter toxicus JCM 9669^{T} (D84127)
Rathayibacter rubneri ZW T2_19^{T} (ON892742)
Rathayibacter oskolensis VKM Ac-2121^{T} (KX758084)
Rathayibacter caricis VKM Ac-1799^{T} (AF159364)
Rathayibacter festucae VKM Ac-1390^{T} (AF159365)
Rathayibacter tritici DSM 7486^{T} (X77438)
Rathayibacter rathayi DSMZ 7485^{T} (X77439)
Rathayibacter tanaceti VKM Ac-2596^{T} (KU891049)
Rathayibacter agropyri CA-4^{T} (KP118964)
Rathayibacter iranicus ICMP 3496^{T} (KT760411)
Nocardioides albus KCTC 9186^{T} (AF004988)
Nocardioides aequoreus SST-45^{T} (AM295338)
Nocardioides marmoraquaticus CBMAI 1089^{T} (JN615437)
Nocardioides aurantiacus BC 361^{T} (Y18629)
Nocardioides scoriae Sco-D01^{T} (FN386750)
Nocardioides marmoriterrae JOS5-1^{T} (KM199639)
Nocardioides marmoriginsengisoli Gsoil 097^{T} (GQ339892)
Nocardioides marmorisolisilvae KIS18-7^{T} (KM199638)
Marmoricola silvestris S20-100^{T} (KP899230)
Nocardioides caldifontis YIM 730233^{T} (MK968450)
Nocardioides pocheonensis Gsoil 818^{T} (GQ339906)
Marmoricola endophyticus 8BXZ-J1^{T} (KY921599)
***Nocardioides terrisoli* YR1^{T} (ON651656)**
Nocardioides mangrovicus 4Q3S-7^{T} (MK063696)
Acidimicrobium ferrooxidans ICP^{T} (U75647)
0.02

Fig. S1. Neighbor-joining tree generated based on the 16S rRNA gene sequences of strains W11^{T}, SW19^{T}, and YR1^{T}. Branching nodes reproduced by both neighbor-joining and maximum-likelihood trees are denoted by filled circles. The numbers at the branching nodes indicate the percentage of 1,000 bootstrap replications (only values >70% are shown). GenBank accession numbers for 16S rRNA gene sequences are provided in parentheses. The scale bar corresponds to 0.02 substitutions per nucleotide position**.**

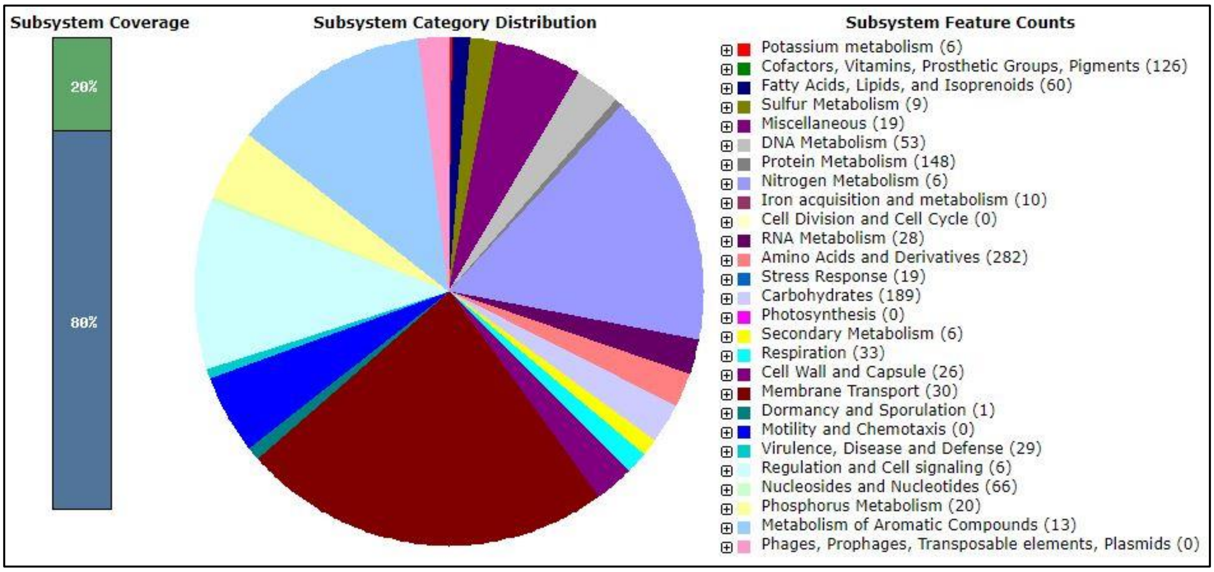

Fig. S2. Genome annotation of strain W11^{T} conducted by RAST (Rapid Annotation using Subsystem Technology) server.

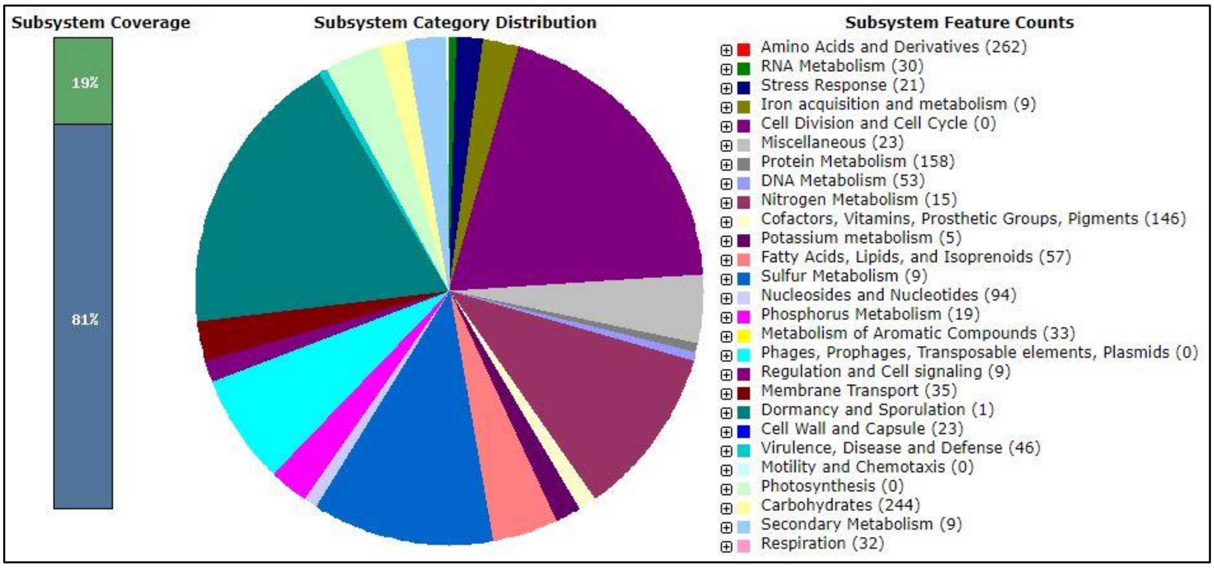

Fig. S3. Genome annotation of strain SW19^T conducted by RAST (Rapid Annotation using Subsystem Technology) server.

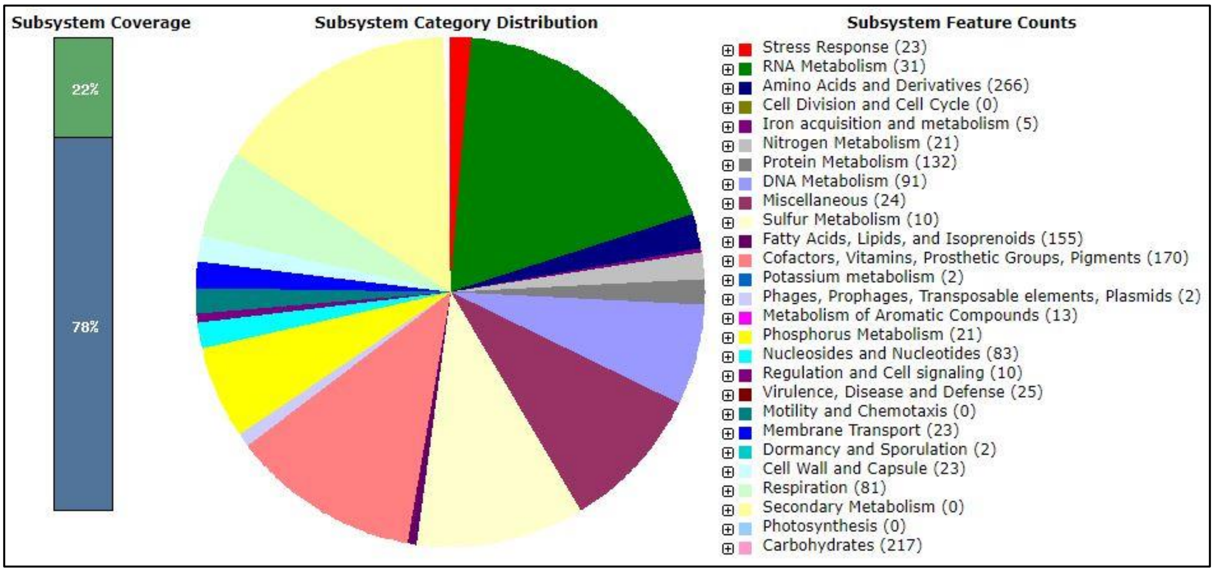

Fig. S4. Genome annotation of strain YR1^{T} conducted by RAST (Rapid Annotation using Subsystem Technology) server.

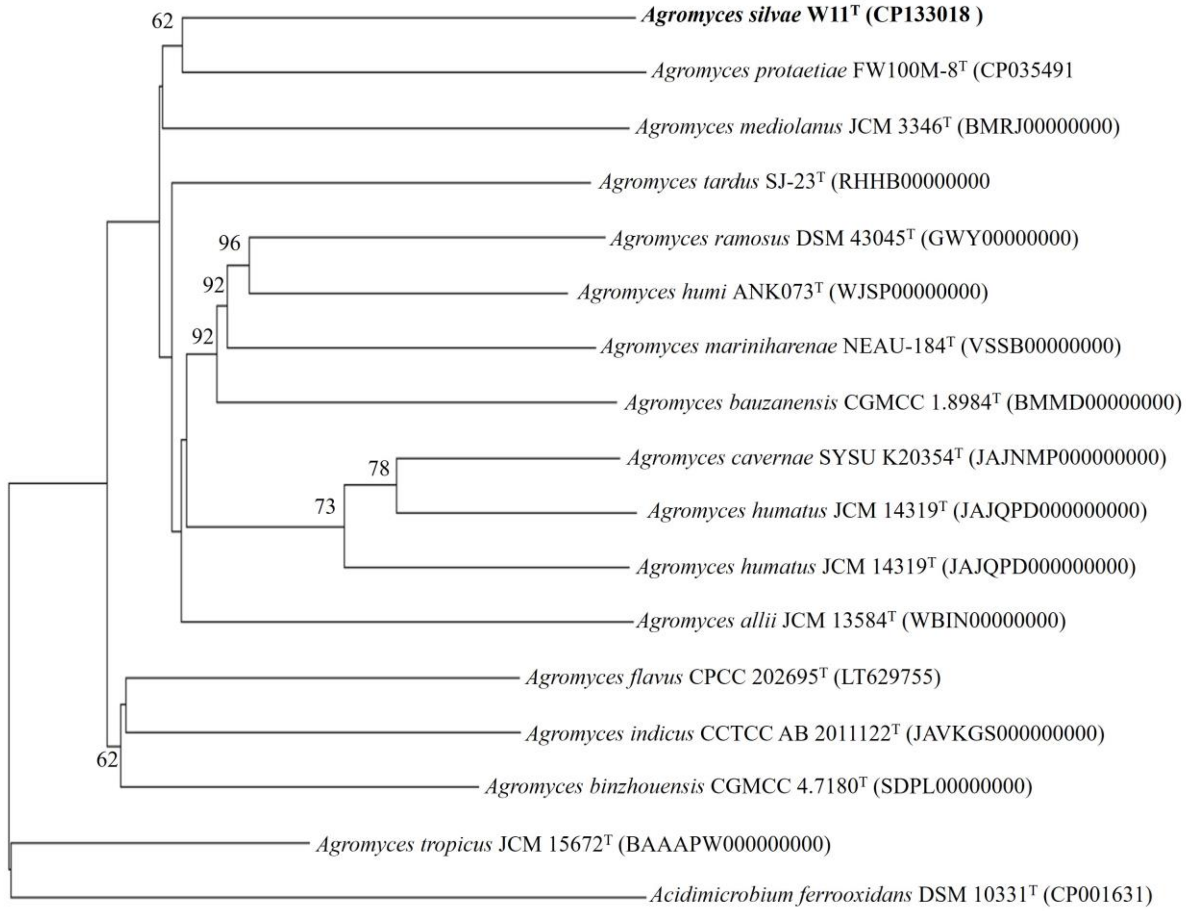

Fig. S5. Phylogenomic tree generated with FastME 2.1.6.1 based on GBDP distances computed from genome data of strain W11^{T} and reference strains. The numbers illustrated at the branches are GBDP pseudo-bootstrap support values from 100 replications.

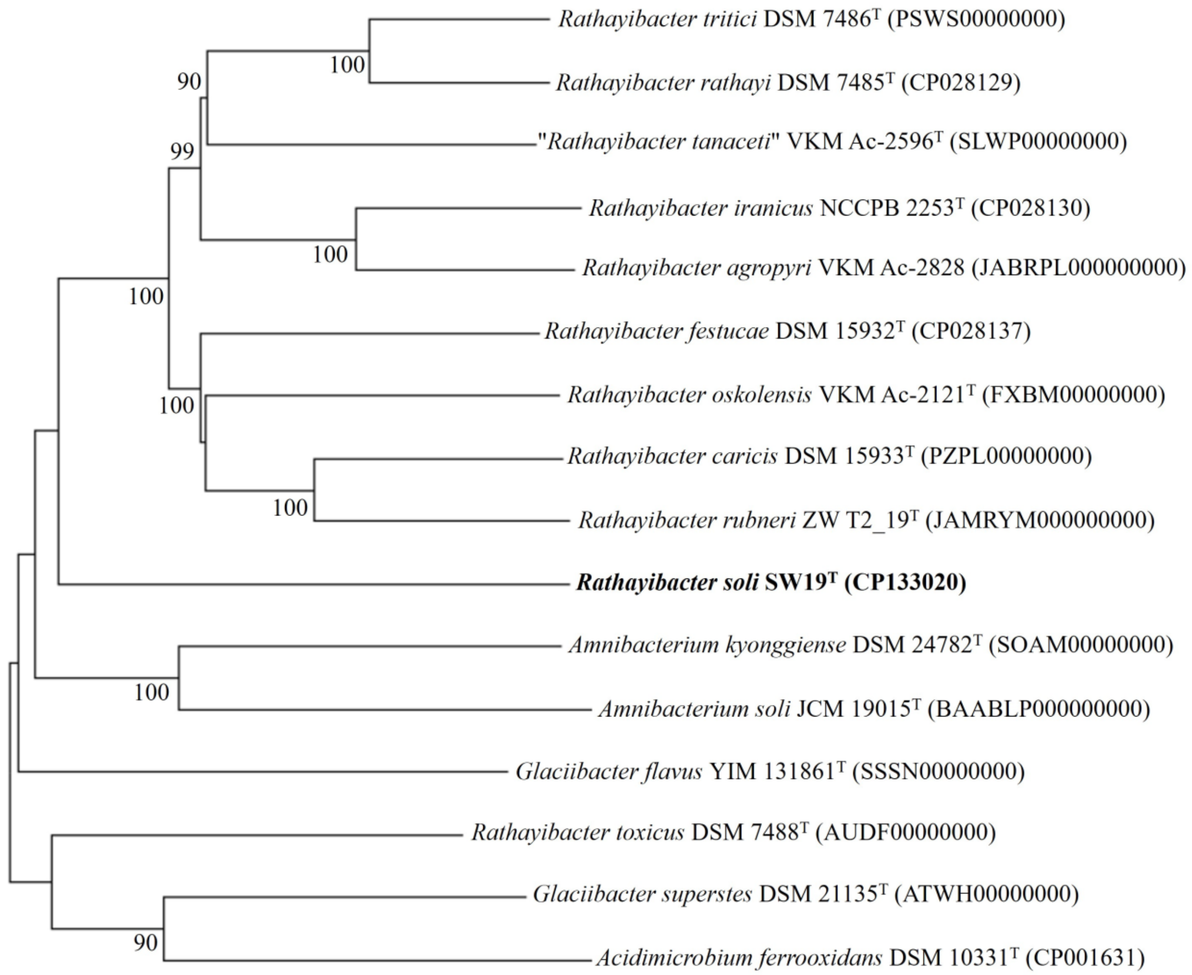

Fig. S6. Phylogenomic tree generated with FastME 2.1.6.1 based on GBDP distances computed from genome data of strain SW19^{T} and reference strains. The numbers illustrated at the branches are GBDP pseudo-bootstrap support values from 100 replications.

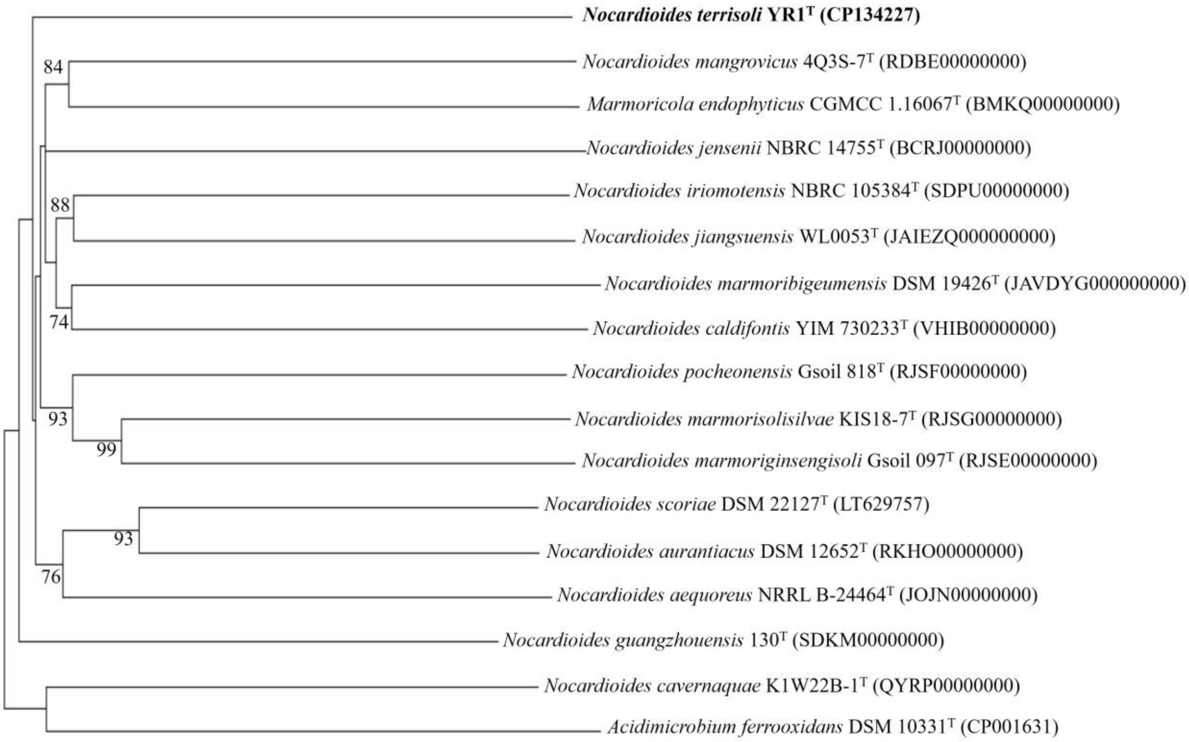

Fig. S7. Phylogenomic tree generated with FastME 2.1.6.1 based on GBDP distances computed from genome data of strain YR1^T and reference strains. The numbers illustrated at the branches are GBDP pseudo-bootstrap support values from 100 replications.

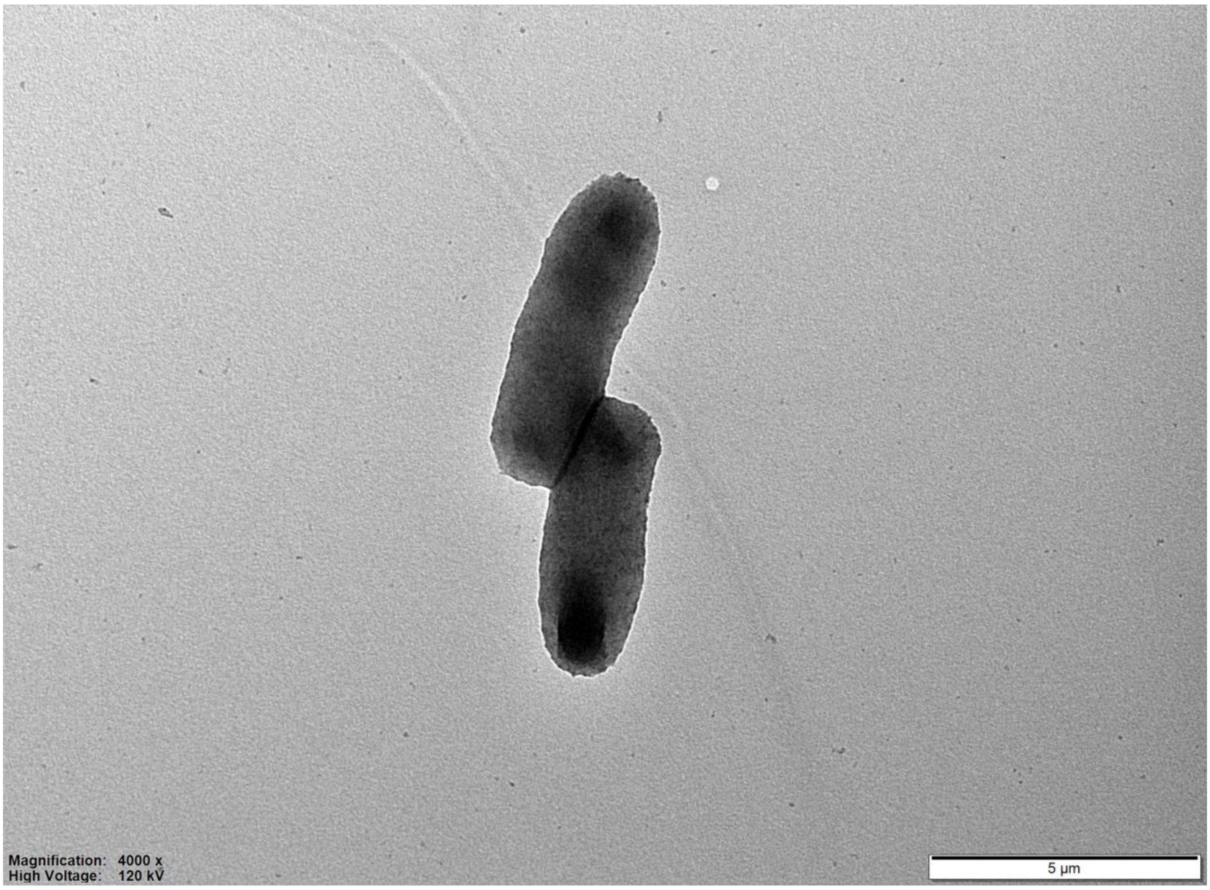

Fig. S8. Transmission electron photomicrograph of strain W11^{T}. Bar, 5.0 µm.

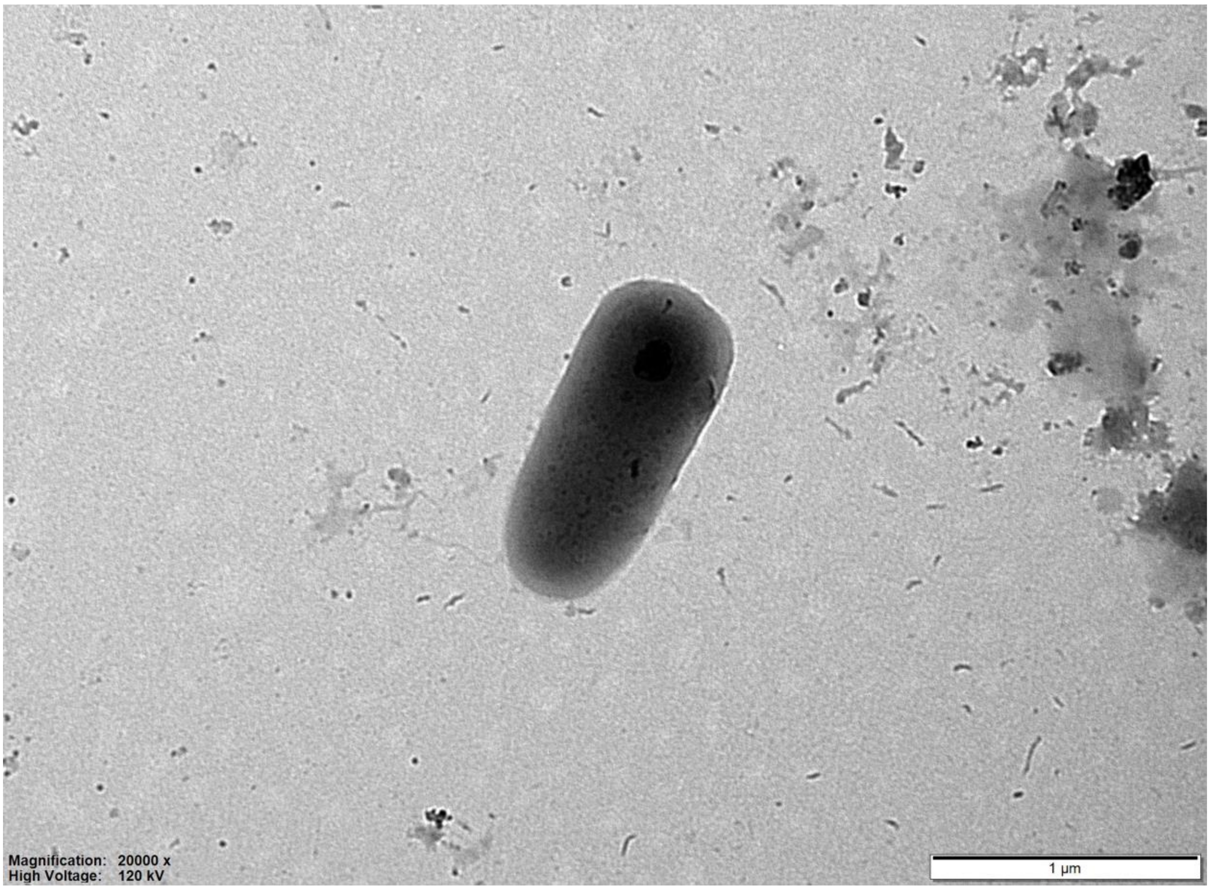

Fig. S9. Transmission electron photomicrograph of strain SW19^T. Bar, 1.0 µm.

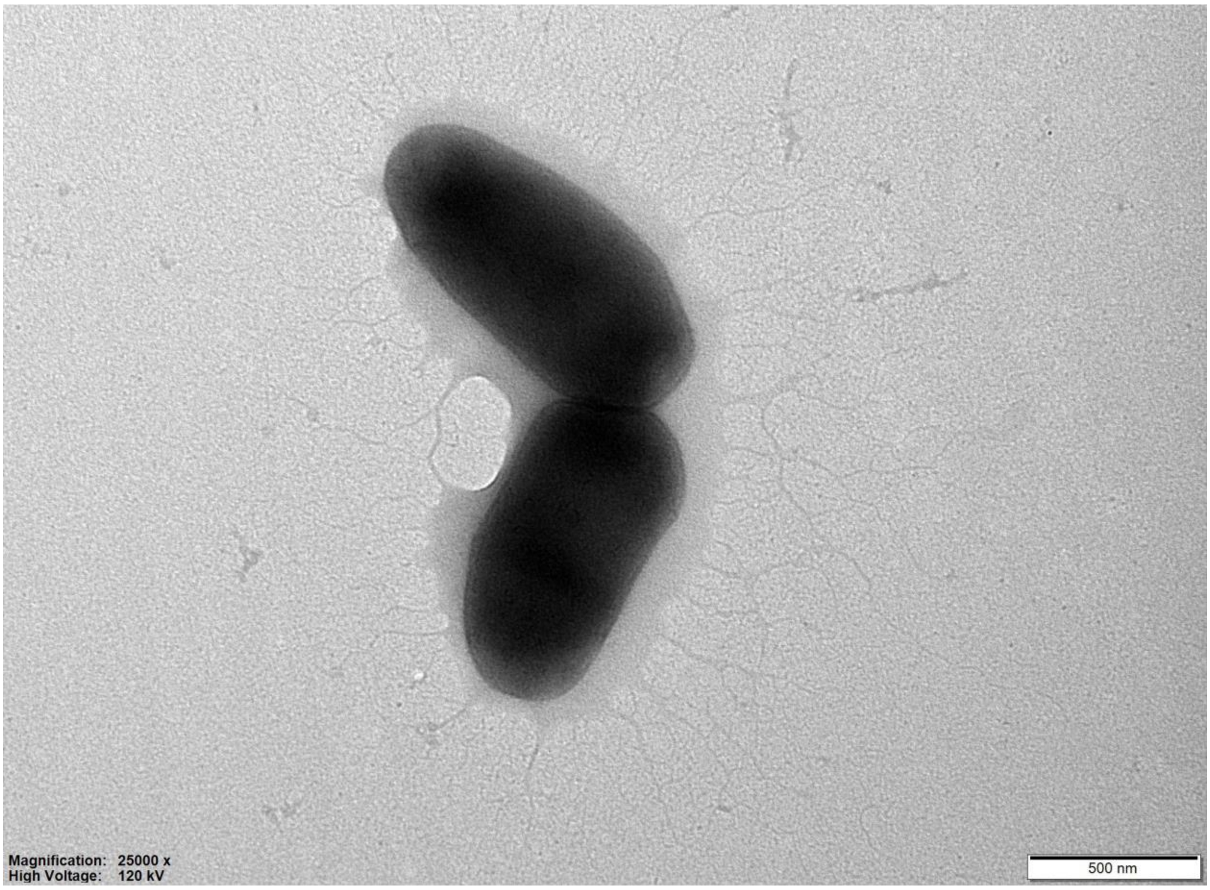

Fig. S10. Transmission electron photomicrograph of strain YR1^{T}. Bar, 500 nm.

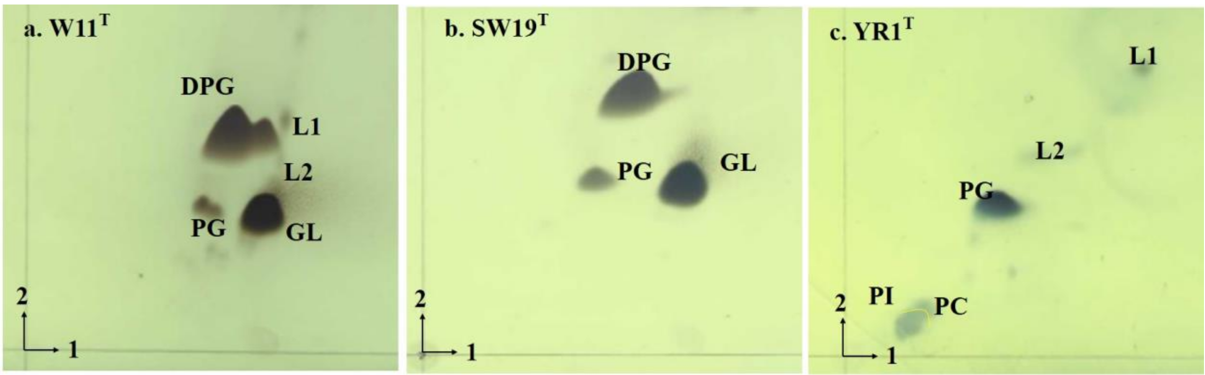

Fig. S11. Thin-layer chromatograms of the polar lipids extracted from strains W11^{T}, SW19^{T}, and YR1^{T}. The components were observed by spraying with 5% molybdophosphoric acid in ethanol and heating at 180°C for 15 min. Abbreviations: PG, phosphatidylglycerol; DPG, diphosphatidylglycerol; PI, phosphatidylinositol; PC, phosphatidylcholine; GL, unidentified glycolipid; and L1-L2, unidentified polar lipids.
